# Effects of Varied Omega-3 Fatty Acid Supplementation on Postpartum Mental Health and the Association between Prenatal Erythrocyte Omega-3 Fatty Acid Levels and Postpartum Mental Health

**DOI:** 10.3390/nu15204388

**Published:** 2023-10-16

**Authors:** Akiko Harauma, Hajime Yoshihara, Yukino Hoshi, Kei Hamazaki, Toru Moriguchi

**Affiliations:** 1 Laboratory for Functional Lipid Science, School of Life and Environmental Science, Azabu University, 1-17-71 Fuchinobe, Chuo, Sagamihara 252-5201, Japan; harauma@azabu-u.ac.jp; 2 Japan Community Health Care Organization, Sagamino Hospital, 1-2-30 Fuchinobe, Chuo, Sagamihara 252-0206, Japan; yhajime6201@yahoo.co.jp; 3 Laboratory of Food and Nutritional Science, Department of Food and Life Science, School of Life and Environmental Science, Azabu University, 1-17-71 Fuchinobe, Chuo, Sagamihara 252-5201, Japan; hoshi@azabu-u.ac.jp; 4 Department of Public Health, Gunma University Graduate School of Medicine, 3-39-22 Showa, Maebashi 371-8511, Japan; kei.hamazaki@gunma-u.ac.jp

**Keywords:** omega-3 fatty acids, α-linolenic acid, EPA, DHA, perilla oil, fish oil, mental, pregnant woman, Edinburgh Postnatal Depression Scale, Mother-to-Infant Bonding Scale

## Abstract

We investigated the postpartum mental health of women who had consumed perilla oil or fish oil containing various omega-3 fatty acids for 12 weeks starting in mid-pregnancy. The association between fatty acids in maternal erythrocytes and mental health risk factors was also examined. Healthy Japanese primiparas in mid-pregnancy (gestational weeks 18–25) were randomly divided into two groups and consumed approximately 2.0 g/day of omega-3 fatty acids in either perilla oil (the ALA dose was 2.4 g/day) or fish oil (the EPA + DHA dose was 1.7 g/day) for 12 weeks. Maternal mental health was assessed using the Edinburgh Postnatal Depression Scale (EPDS) as the primary measure and the Mother-to-Infant Bonding Scale (MIBS) as the secondary measure. Data from an observational study were used as a historical control. Maternal blood, cord blood, and colostrum samples were collected for fatty acid composition analysis. In addition, completers of the observational studies were enrolled in a case–control study, wherein logistic regression analysis was performed to examine the association between maternal fatty acids and EPDS score. The proportion of participants with a high EPDS score (≥9) was significantly lower in the perilla oil group (12.0%, *p* = 0.044) but not in the fish oil group (22.3%, *p* = 0.882) compared with the historical control (21.6%), while the proportions between the former groups also tended to be lower (*p* = 0.059). No marked effect of omega-3 fatty acid intake was observed from the MIBS results. In the case–control study of the historical control, high levels of α-linolenic acid in maternal erythrocytes were associated with an EPDS score of <9 (odds ratio of 0.23, 95% confidence interval: 0.06, 0.84, *p* = 0.018 for trend). The results of this study suggest that consumption of α-linolenic acid during pregnancy may stabilize postpartum mental health.

## 1. Introduction

For women, pregnancy and childbirth are significant life events accompanied by changes in hormone activity, anxiety about childcare, and changes in life routines and health behaviors. During these events, perinatal mood instability is common, and this can lead to baby blues and postnatal depression. In particular, anxiety factors are increased when mothers are primiparous and/or older [1]. The mental health of pregnant women has a great impact on the quality of childrearing and the establishment of mother–child bonding [2]; in serious cases, parenting may be inadequate, which may negatively influence the child’s development [3]. Moreover, during the perinatal period, mothers tend to avoid taking medication as much as possible in order to avoid adverse effects on their babies. Thus, it would be desirable to stabilize the mental health of expectant mothers through their daily diet and living environment.

Omega-3 fatty acids are important for health; however, they are not synthesized in the body and must be consumed directly from the diet. Major omega-3 fatty acids include α-linolenic acid (ALA), eicosapentaenoic acid (EPA), and docosahexaenoic acid (DHA). ALA, which is contained in perilla oil and linseed oil, is metabolized in vivo and partially converted to EPA and DHA. In addition, EPA and DHA can be obtained by consuming fish oil [4]. It has been reported that intake of EPA reduces the risk of cardiovascular disease and suppresses inflammation [5,6], while intake of DHA contributes to brain function as well as the development and function of the eyes [7,8]. For these reasons, many studies have focused on seafood that is rich in EPA and DHA. For example, one study reported an inverse relationship between seafood consumption and the risk of postpartum depression [9]. In pregnant women, DHA is supplied from the mother to the fetus for growth and development, so it is thought that expectant mothers are at increased risk of depression due to DHA deficiency [10]. Although many studies on omega-3 fatty acids have assumed that seafood consumption is higher in Japan than in other countries, seafood consumption peaked in Japan in 2001 and has been decreasing every year since, making EPA and DHA deficiency in individuals of reproductive age a cause for concern [11]. Indeed, the beneficial effects of seafood and omega-3 fatty acid intake on perinatal depression was reported in Japan [12,13,14]. In an animal study, female mice raised on a diet deficient in omega-3 fatty acids showed delayed development of maternal behavior, and after giving birth, they tended to neglect the newborn pups. [15]. In another study, dams raised on a diet deficient in omega-3 fatty acids had low brain DHA and oxytocin levels and had difficulties continuing maternal behavior after giving birth [16]. These findings suggest that stabilizing maternal mental health during the perinatal period through nutritional support might help to improve the mental health of pregnant and parturient women and increase the birth rate.

The present study examined the effects of omega-3 fatty acids on perinatal mental health by examining the degree of psychological distress 1 month after giving birth using the Edinburgh Postnatal Depression Scale (EPDS) as well as the degree of attachment with their children using the Mother-to-Infant Bonding Scale (MIBS) in mothers who consumed omega-3 fatty acids in either perilla oil or fish oil. In addition, a case–control study was conducted to evaluate the association between prenatal erythrocyte fatty acid levels and postpartum mental health.

## 2. Materials and Methods

### 2.1. Participants

**Figure 1:**
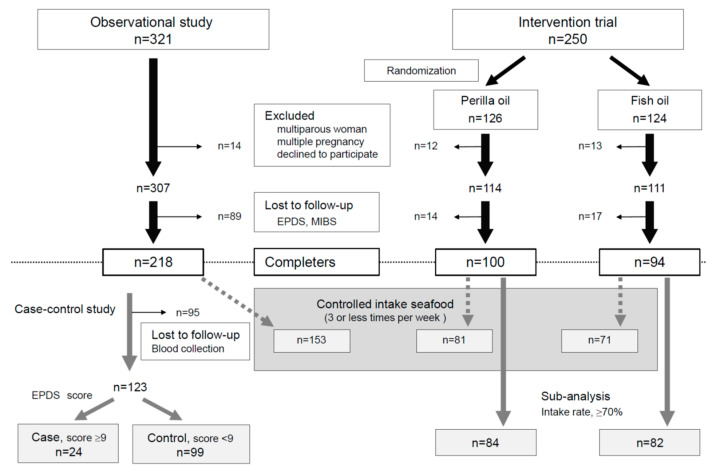
Flowchart of participant screening and enrollment.

The Perinatal Medical Center, Sagamino Hospital, Japan Community Health Care Organization, recruited the participants for this study, and Azabu University performed tests of the samples and analyzed the data. A total of 321 participants were recruited for the observational study between November 2015 and June 2017 and 250 for the interventional study between July 2017 and April 2019. These were 2 different sets of participants, and none of those in the RCT were included in the observational study (Figure 1).

This was a double-blind, parallel-group comparison study wherein participants were randomly assigned to two groups receiving omega-3 fatty acids in either perilla oil or fish oil (the recruited period was 21 months for July 2017 to April 2019), and the two abovementioned groups plus a group of participants in the observational study (historical control; the recruited period was 19 months for November 2015 and June 2017) were compared (Figure 1).

All subjects gave their informed consent for inclusion before they participated in the study. This study was approved by the Human Research Ethics Committee of Azabu University (No. 083, 102) and was registered in the University Hospital Medical Information Network (UMIN) Center Clinical Trial Registry (UMIN000027170).

### 2.2. Study Design

Participants were healthy primiparous women aged 19–45 years; multiparous women, those with multiple pregnancies, those who declined to participate in the study, and those who did not submit the 1-month postpartum questionnaire were excluded.

Intervention groups received omega-3 fatty acids starting in mid-pregnancy (gestational weeks 18–25) for 12 weeks. The perilla oil group received 4.0 g/day perilla oil containing 2.4 g/day ALA, while the fish oil group received 5.2 g/day sardine oil containing 1.3 g/day EPA and 0.4 g/day DHA. Perilla oil was provided by Ota Oil Co., Ltd. (Okazaki, Japan), and fish oil was provided by Nissui Corporation (Tokyo, Japan); both products were individually packaged without any labels.

The mental health of participants was assessed using the Kessler Psychological Distress Scale (K6) [17] at mid-pregnancy (start of the study) and immediately after giving birth (i.e., within 5 days after giving birth), as well as at 1 month after giving birth, using the EPDS and the MIBS [18,19]. In addition, maternal blood was collected in early pregnancy (around gestational week 12), mid-pregnancy (around gestational week 28), late pregnancy (around gestational week 36), and the day after giving birth, while cord blood and colostrum (milk produced within 5 days after giving birth) were collected for fatty acid composition analysis.

In the case study, data from the observational study were divided into two groups (cases with an EPDS score ≥ 9 and controls with an EPDS score < 9). Controls were divided into tertile groups according to fatty acid level, and logistic regression analysis was performed to obtain odds ratios and 95% confidence intervals.

### 2.3. Statistical Analysis

Participant characteristics were compared by *t*-test or Fisher’s Exact test. Comparisons of the primary outcome (EPDS) and a secondary outcome (MIBS) were performed by Fisher’s Exact test. Changes in fatty acids in biological samples collected during the study period were examined by two-way analysis of variance with the Tukey multiple comparison test.

Differences in levels of various types of fatty acids in maternal erythrocytes a day after giving birth in the case–control study were compared by the Mann–Whitney *U* test. Logistic regression analysis was performed to obtain crude odds ratios and 95% confidence intervals, and then multivariable logistic regression was performed with adjustment for the following five confounding factors: age, pre-pregnancy body mass index, smoking status (never smoker, stopped before pregnancy, stopped upon pregnancy, current smoker), alcohol intake (never drinker, stopped before pregnancy, stopped upon pregnancy, current drinker), and taking supplements (yes/no). Category numbers were assigned to tertiles according to fatty acid level and examined as continuous variables to test for trends.

Data were analyzed using SPSS Statistics 25 (IBM Corp., Armonk, NY, USA). A *p*-value of 0.05 or lower was considered statistically significant.

### 2.4. Fatty Acid Analysis

Erythrocytes (obtained by centrifugation of maternal blood and cord blood) and colostrum were stored at −80 °C until analysis. The sample (100 µL) was added to 2 mL methanol:hexane (4:1) solvent containing 50 µg/mL butylhydroxytoluene. The transmethylation method developed by Lepage and Roy was used [20]. Fatty acid methyl esters were analyzed using the methods of Masood et al. [21]. A standard fatty acid mixture (NuChek Prep 462; Elysian, MN, USA) was used to verify the identification and assignment of retention times. Each fatty acid was expressed as % of total fatty acids.

## 3. Results

### 3.1. Disposition and Demographics

The recruitment period of the observation study was from November 2015 to June 2017. Among the 321 participants initially enrolled, 14 were excluded because they were multiparous or with multiple pregnancies or declined to participate in the study. Another 89 were excluded because they did not participate in the mental health assessment. The data from the remaining 218 participants were analyzed. The recruitment period of the interventional study was from July 2017 to April 2019. A total of 250 participants were enrolled and then randomly assigned to the perilla oil group (*n* = 126) or the fish oil group (*n* = 124); 12 were eliminated from the perilla oil group and 13 from the fish oil group because they were multiparous or with multiple pregnancies or declined to participate in the study. Another 14 and 17 were eliminated from the perilla oil group and the fish oil group, respectively, because they did not participate in the mental health assessment. The data from the remaining 100 participants in the perilla oil group and the remaining 94 in the fish oil group were analyzed (Figure 1). The detailed characteristics of participants in the interventional study are shown in Table 1. There were no differences in characteristics between the excluded participants (historical control; *n* = 89, perilla oil; *n* = 14, fish oil; *n* = 17) and the participants used in the experiment.

In the case–control study, 95 participants without blood samples were eliminated, and the remaining 123 participants were divided into cases (*n* = 24, EPDS score ≥ 9) and controls (*n* = 99, EPDS score < 9) (Figure 1). The characteristics of cases and controls were compared (Table 2). There were no differences in characteristics between the excluded participants (*n* = 95) and the participants used in the experiment.

Because the participants for both the interventional and case–control studies were recruited by the same center, and the recruitments for those studies were carried out in sequence, the basic characteristics of participants in both studies were largely the same.

### 3.2. Outcomes

Assessment of the degree of psychological distress at the time of recruitment (mid-pregnancy) and immediately after giving birth showed that the proportions of participants with a high K6 score (≥13) were very low (<3%) (Table 3). The proportions of participants with a high EPDS score (≥9) 1 month after giving birth were 21.6% in the historical control group, 12.0% in the perilla oil group, and 22.3% in the fish oil group; the proportion was significantly lower in the perilla oil group compared with the historical control group (*p* = 0.044), and the proportion with a high EPDS score was nearly significantly lower in the perilla vs. the fish oil group (*p* = 0.059; Table 4). The proportions of participants with an MIBS score ≥ 3 or ≥5 were 24–30% and 10–14%, respectively and did not differ markedly among the three groups (Table 4). This effect did not change, even after controlling for participants with a high seafood intake (four or more times per week). A high EPDS score in the historical control was 22.2% (*n* = 153), that in the perilla oil group was 11.1% (*n* = 81, vs. historical control: *p* = 0.050), and that in the fish oil group was 22.5% (*n* = 71, vs. perilla: *p* = 0.079) (Table A1). A subanalysis was conducted to confirm the influence of each participant’s omega-3 oil intake status on mental health scores. Subanalysis of participants whose self-assessed consumption rate of omega-3 fatty acids over 12 weeks was ≥70% (assessment options: Perfect, ≥90%, ≥70%, <60%, almost none) showed an even lower proportion of those with a high EPDS score (9.5%) in the perilla oil group (*n* = 84, vs. historical control: *p* = 0.019), while that in the fish oil group (23.2%, *n* = 82) was similar (vs. perilla: *p* = 0.021; Table A2).

**Figure 2:**
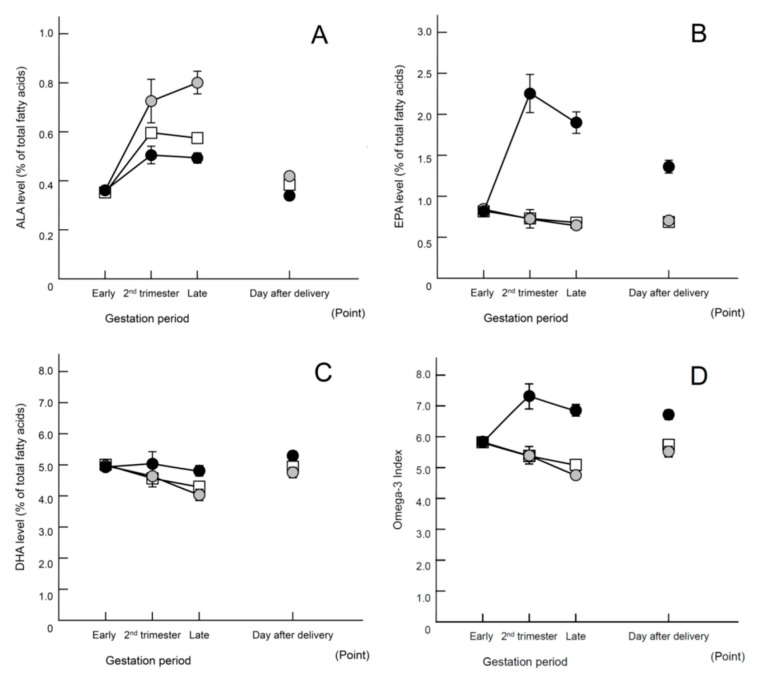
Changes in fatty acids in maternal erythrocytes (% of total fatty acids). Data are presented as means ± SEM. Open square, historical control; gray circle, perilla oil; closed circle, fish oil. (**A**) α-Linolenic acid (ALA); (**B**) eicosapentaenoic acid (EPA); (**C**) docosahexaenoic acid (DHA); (**D**) omega-6/omega-3 ratio. Statistical analysis using two-way analysis of variance yielded the following results: (**A**) Group effects: F (2, 1166) = 34.935, *p* < 0.01. Tukey’s test indicated the following significant differences between all groups: *p* < 0.01. Interaction effect: F (6, 1166) = 12.649, *p* < 0.001. (**B**) Group effects: F (2, 1166) = 244.447, *p* < 0.01. Tukey’s test indicated the following significant differences: historical control, *p* < 0.01; perilla, *p* < 0.01 compared with fish oil group. Interaction effect: F (6, 1166) = 47.553, *p* < 0.01. (**C**) Group effects: F (2, 1166) = 13.016, *p* < 0.01. Tukey’s test indicated the following significant differences: historical control, *p* < 0.01; perilla oil, *p* < 0.01 compared with the fish oil group. Interaction effect: F (6, 1166) = 4.486, *p* < 0.01. (**D**) Group effects: F (2, 1166) = 81.651, *p* < 0.01. Tukey’s test indicated the following significant differences: historical control, *p* < 0.01; perilla oil, *p* < 0.01 compared with fish oil group. Interaction effect: F (6, 1166) = 18.405, *p* < 0.01. Regarding the omega-3 oil intake status at blood collection in the Late, 32% of the participants in perilla oil group and 46% of the participants in fish oil group had completed supplementation.

Analysis of changes in fatty acids in maternal erythrocytes showed increases in ALA levels in the perilla oil group as well as increases in EPA and DHA levels in the fish oil group, confirming that those groups consumed oils containing the corresponding omega-3 fatty acids (Figure 2A–C). The omega-3 index was markedly increased only in the fish oil group (Figure 2D). Table 5 shows the fatty acid compositions in detail. Even though the intervention (i.e., consumption of oils containing omega-3 fatty acids) was completed a month before giving birth, the levels of DHA remained high in the erythrocytes of cord blood samples in the fish oil group (Table A3 and Table A4), suggesting that a preferential supply of omega-3 fatty acids to newborn babies was attributable to the consumption of fish oil during mid-pregnancy.

### 3.3. Case–Control Study (Observational Study)

Fatty acid compositions in maternal erythrocytes the day after giving birth showed significantly lower ALA levels in cases compared with controls (*p* = 0.039, Table 6). Logistic regression analysis of tertile groups according to fatty acid level was performed to examine the degrees of psychological distress. A significant crude odds ratio was shown only in the highest tertile according to ALA level compared with the lowest tertile (crude odds ratio of 0.29, 95% confidence interval: 0.09, 0.96, *p* = 0.030 for trend). Similarly, the multivariable model adjusted for five confounding factors (age, pre-pregnancy body mass index, smoking status, alcohol intake, and taking supplements) showed a significant odds ratio in the highest tertile according to ALA level compared with the lowest tertile (a multivariable odds ratio of 0.23, 95% confidence interval: 0.06, 0.84, *p* = 0.018 for trend, Table 7). Taken together, the results suggest that high maternal erythrocyte ALA levels may help to mitigate psychological distress.

## 4. Discussion

In a global survey, it was reported that 10–20% of pregnant women experience postpartum depression [22]. This percentage increases in primiparous women because they have many anxiety factors associated with childbirth, childrearing, and other events, all of which are new to them [1]. The present study examined the effects of omega-3 fatty acids on perinatal mental health in primiparous women who are prone to mental instability. There are several reports of trials involving EPA and DHA, but few involving ALA [23]. Although the contribution of ALA is generally thought to be low, we considered it important to investigate whether this is indeed the case.

Of the primiparous women recruited into this study over 3.5 years (between November 2015 and April 2019), approximately 3% had a high K6 score (≥13), indicating that this population contained very few individuals with mental instability during pregnancy. The omega-3 index of maternal erythrocytes around gestational week 12 in this study was as low as in European and North American populations (>4–6%) (Table 5). Schuchardt et al. reported the following omega-3 index values of erythrocytes as long-term biomarkers: desirable, >8%; moderate, >6–8%; low, >4–6%; very low, ≤4% [24]. The proportion of those with a high EPDS score (≥9) in the postpartum period was 21.6% in the observational study (subjects in the first half of recruitment), indicating that this population was a typical primiparous population in Japan. However, analysis of the interventional study (subjects in the second half of recruitment) showed that the proportion of those with a high EPDS score decreased to 12.0% in the perilla oil group (*p* = 0.044, Table 4). This effect did not change even after controlling for participants with a high seafood intake (four or more times per week) (Table A1). Also, this effect was more prominent in the group having a higher consumption rate (Table A2). It would be a finding of great importance if consumption of ALA during pregnancy stabilizes postpartum mental health.

The possibility that EPA and DHA prevent or mitigate postpartum depression has been demonstrated previously [23]. Such benefits were more likely to be observed in people with severely depressed status (EPDS score ≥ 12 or Hamilton Rating Scale for Depression ≥ 20) [25]. Participants in the present study were healthy pregnant women, judged by K6 score, and this may explain why the effect of fish oil was not observed in this study. This raises the question of why the proportion of those with a high EPDS score decreased only in the perilla oil group. We do not think that this occurred by chance because the case–control study of participants in the observational study showed that maternal erythrocyte ALA level was associated with a high EPDS score. A 10-year follow-up study of middle-aged and older women demonstrated that the risk of depression was not associated with intake of EPA plus DHA but was inversely associated with ALA intake; this association was stronger in women with low linoleic acid intake [26]. An inverse correlation between fear of breast cancer recurrence and blood ALA level has also been reported [27]. It is likely that the results of the present study show the mitigation of a vague anxiety about childbirth and childrearing but not a pathologic mental disorder. Thus, a more detailed assessment of the degree of anxiety using the State–Trait Anxiety Inventory and the Manifest Anxiety Scale is necessary.

The Omega-6/Omega-3 ratio is treated as one of the important items for detecting the effects of PUFA [28,29]. In the present study, fish oil intervention clearly decreased the Omega-6/Omega-3 ratio, whereas perilla oil intervention did not show any change. However, only the perilla oil intervention improved mental health scores, suggesting that changes in the Omega-6/Omega-3 ratio do not affect pregnant women’s emotions. Furthermore, it was suggested that metabolites such as oxylipin, which are unique to perilla oil, may be contributing to the improvement in mental health scores due to perilla oil intervention.

Differences in ALA metabolism [30] are likely to contribute to the effect seen in the perilla oil group in this study. During pregnancy, maternal DHA is preferentially distributed via transport proteins to the fetus for its development [31]. Indeed, levels of EPA, DHA, and docosapentaenoic acid *n*-3 in erythrocytes of cord blood were high in the fish oil group, suggesting they were supplied to the fetus [32]. However, DHA levels in erythrocytes of cord blood were significantly lower in the perilla oil group than in the other two groups. DHA synthesized from ALA appears in blood at a different time compared with DHA that has been ingested [33]. This difference may have been responsible for the accumulated effects on the mothers themselves rather than the supply of DHA to the fetus. The omega-3 index of erythrocytes indicated that the participants were DHA-deficient. It is considered that DHA metabolized from ALA was preferentially accumulated in the maternal brain instead of the fetus. Therefore, in the future, it will be necessary to measure DHA levels in the brain.

Reduced intake of omega-3 fatty acids is a global phenomenon, and Japan is no exception. In fact, there has been a marked decline in seafood consumption in Japan [11]. Considering that it takes a long time to restore DHA in the body [34], we believe that it is necessary to sound the alarm, even in areas where seafood consumption was previously commonplace. No effect on maternal mental health was found in the fish oil group. However, given the changes observed in fatty acid compositions in maternal blood and cord blood, fish oil is expected to have beneficial effects on newborn babies [35]. Adequate supplementation of EPA and DHA in early pregnancy reduces the risk of preterm birth, and the resulting continuation of pregnancy leads to the prevention of premature births [36]. In the future, we would like to analyze the relationship of maternal intake of omega-3 fatty acids with the growth and development of newborn babies.

### Limitations

A placebo control was not included in the interventional study because of humanitarian considerations. The State–Trait Anxiety Inventory and the Manifest Anxiety Scale were not used in addition to EPDS. Socio-economic status was not assessed. The omega-3 fatty acid contents in the consumed oils were not precisely adjusted. Analysis of blood fatty acids alone did not depict a complete picture of the changes in the brain. The results obtained here may be limited to pregnant women in such regions since Japan is a region where the intake of omega-3 fatty acids is generally higher than in other countries.

## 5. Conclusions

ALA intake during pregnancy may stabilize maternal mental health at 1 month after giving birth. In addition, high levels of maternal blood ALA were suggested to be associated with mitigation of psychological distress.

## Figures and Tables

**Table 1:** Participant characteristics.

	Observational Study	Intervention Trial				
	Historical Control ^1^	Perilla Oil Group ^1^	Fish Oil Group ^1^	^a^ *p*	^b^ *p*	^c^ *p*
	*n* = 218	*n* = 100	*n* = 94			
Age (year)	32.5 ± 4.9	31.8 ± 5.0	33.1 ± 5.8	0.202	0.363	0.084
Height (cm)	158.3 ± 5.2	158.0 ± 5.8	158.1 ± 5.0	0.666	0.783	0.891
BMI (pre-pregnancy)	21.3 ± 3.1	21.3 ± 2.7	21.8 ± 3.6	0.933	0.258	0.281
BMI (post-pregnancy)	25.4 ± 2.9	25.4 ± 2.8	26.0 ± 3.3	0.869	0.099	0.202
Gestational weeks	39.1 ± 1.2	39.2 ± 1.1	39.1 ± 1.3	0.328	0.817	0.553
Smoking status				0.982	0.549	0.777
Never smoker	149	72	71			
Stopped before pregnancy	42	18	13			
Stopped upon pregnancy	20	9	8			
Current smoker	2	1	0			
Alcohol intake				0.843	0.953	0.914
Never drinker	10	6	4			
Stopped before pregnancy	86	41	39			
Stopped upon pregnancy	117	53	49			
Current drinker	0	0	0			
Taking supplement ^2^						
yes/no	146/68	69/31	68/26	1.000	0.504	0.639
omega-3 supplement	4	1	5			

^1^ Mean ± SD; *p*-value: *t*-test for continuous variables and Fisher’s Exact test for categorical variables; ^a^ *p*: Historical control vs. perilla oil; ^b^ *p*: Historical control vs. fish oil; ^c^ *p*: Perilla oil vs. fish oil. ^2^ More than 90% of the supplements were folic acid and/or iron.

**Table 2:** Case–control participant characteristics.

	Cases ^1^	Controls ^1^	*p*
	*n* = 24	*n* = 99	
Age (year)	31.5 ± 4.0	32.6 ± 5.2	0.370
Height (cm)	157.8 ± 5.4	158.7 ± 5.1	0.440
BMI (pre-pregnancy)	21.6 ± 4.2	21.3 ± 3.2	0.703
BMI (post-pregnancy)	25.5 ± 3.4	25.4 ± 3.0	0.837
Gestational weeks	38.8 ± 1.3	39.3 ± 1.2	0.069
Smoking status			0.187
Never smoker	18	61	
Stopped before pregnancy	2	24	
Stopped upon pregnancy	3	10	
Current smoker	1	1	
Alcohol intake			0.563
Never drinker	2	5	
Stopped before pregnancy	7	38	
Stopped upon pregnancy	15	53	
Current drinker	0	0	
Taking supplement ^2^			
yes/no	17/7	62/35	0.635
omega-3 supplement	0	1	

^1^ Mean ± SD; *p*-value: *t*-test for continuous variables and Fisher’s Exact test for categorical variables; Cases were an EPDS score ≥ 9, and controls were an EPDS score < 9. ^2^ More than 95% of the supplements were folic acid and/or iron.

**Table 3:** Mean (SD) Kessler Psychological Distress Scale score.

			Observational Study	Intervention Trial	
			Historical Control	Perilla Oil Group	Fish Oil Group
Middle pregnancy		*n*	215	100	94
(weeks 18–25)	Mean ± SD		3.4 ± 3.6	3.5 ± 3.3	2.7 ± 2.8
	K6 ≥ 13	% (*n*)	2.8 (6)	3.0 (3)	0 (0)
Day after giving birth		*n*	182	97	94
	Mean ± SD		3.7 ± 2.9	3.9 ± 3.0	3.1 ± 2.9
	K6 ≥ 13	% (*n*)	2.2 (4)	2.1 (2)	1.1 (1)

**Table 4:** Mean Edinburgh Postnatal Depression Scale and Mother-to-Infant Bonding Scale scores.

			Observational Study	Intervention Trial		^a^ *p*	^b^ *p*	^c^ *p*
			Historical Control	Perilla Oil Group	Fish Oil Group			
One month after childbirth		*n*	218	100	94			
EPDS	Mean ± SD		5.6 ± 4.0	5.4 ± 3.3	5.4 ± 3.9			
	score ≥ 9	% (*n*)	21.6 (47)	12.0 (12)	22.3 (21)	0.044	0.882	0.059
MIBS	Mean ± SD		1.9 ± 2.0	1.8 ± 2.0	2.0 ± 2.3			
	score ≥ 3	% (*n*)	29.8 (65)	24.0 (24)	28.7 (27)	0.346	0.893	0.515
	score ≥ 5	% (*n*)	11.0 (24)	10.0 (10)	13.8 (13)	0.848	0.567	0.507

*p*-value: Fisher’s Exact test for categorical variables; ^a^ *p*: Historical control vs. perilla oil; ^b^ *p*: Historical control vs. fish oil; ^c^ *p*: Perilla oil vs. fish oil. EPDS, Edinburgh Postnatal Depression Scale; MIBS, Mother-to-Infant Bonding Scale; SD, standard deviation.

**Table 5:** Fatty acid composition of maternal erythrocytes.

		Observational Study				Intervention Trial							
		Historical Control				Perilla Oil Group				Fsh Oil Group			
		Early Pregnancy	2nd Trimester	Late Pregnancy	Day after Delivery	Early Pregnancy	2nd Trimester	Late Pregnancy	Day after Delivery	Early Pregnancy	2nd Trimester	Late Pregnancy	Day after Delivery
Fatty Acids		*n* = 151	*n* = 76	*n* = 129	*n* = 123	*n* = 83	*n* = 18	*n* = 64	*n* = 57	*n* = 80	*n* = 21	*n* = 67	*n* = 62
Total saturated fatty acids		40.09 ± 0.17	37.88 ± 0.24	38.26 ± 0.23	41.02 ± 0.19	40.76 ± 0.22	39.86 ± 0.60	38.66 ± 0.32	41.78 ± 0.23	40.28 ± 0.26	38.75 ± 0.55	39.98 ± 0.33	42.57 ± 0.19
Total mono-unsaturated fatty acids		21.13 ± 0.12	23.78 ± 0.19	24.78 ± 0.19	23.9 ± 0.16	20.82 ± 0.17	21.31 ± 0.32	24.16 ± 0.23	22.96 ± 0.22	21.07 ± 0.19	22.03 ± 0.40	23.34 ± 0.26	22.67± 0.18
ω-6 polyunsaturated fatty acids													
Linoleic acid (LA)	18:2n-6	17.41 ± 0.24	20.41 ± 0.35	19.62 ± 0.35	15.24 ± 0.26	17.16 ± 0.33	18.93 ± 0.88	19.57 ± 0.46	14.67 ± 0.27	17.71 ± 0.36	18.71 ± 0.84	17.20 ± 0.45	13.64 ± 0.29
Arachidonic acid (ARA)	20:4n-6	9.65 ± 0.10	7.27 ± 0.13	6.97 ± 0.14	8.35 ± 0.11	9.79 ± 0.13	8.09 ± 0.31	7.17 ± 0.17	8.74 ± 0.13	9.71 ± 0.15	7.62 ± 0.29	6.96 ± 0.16	8.10 ± 0.12
Docosapentaenoic acid n-6 (DPAn-6)	22:5n-6	0.27 ± 0.01	0.27 ± 0.01	0.26 ± 0.01	0.34 ± 0.01	0.34 ± 0.01	0.38 ± 0.03	0.32 ± 0.01	0.37 ± 0.01	0.34 ± 0.01	0.28 ± 0.02	0.26 ± 0.01	0.29 ± 0.01
Total ω-6 polyunsaturated fatty acids		29.91 ± 0.20	30.36 ± 0.26	29.20 ± 0.22	26.67 ± 0.17	30.05 ± 0.24	30.28 ± 0.58	29.49 ± 0.32	26.70 ± 0.17	30.51 ± 0.24	28.81 ± 0.65	26.53 ± 0.32	24.49 ± 0.24
ω-3 polyunsaturated fatty acids													
α-Linolenic acid (ALA)	18:3n-3	0.36 ± 0.01	0.59 ± 0.02	0.57 ± 0.02	0.38 ± 0.01	0.37 ± 0.02	0.73 ± 0.09	0.81 ± 0.05	0.43 ± 0.02	0.37 ± 0.01	0.51 ± 0.04	0.50 ± 0.02	0.34 ± 0.01
Eicosapentaenoic acid (EPA)	20:5n-3	0.83 ± 0.03	0.78 ± 0.05	0.70 ± 0.03	0.71 ±0.03	0.85 ± 0.05	0.73 ± 0.11	0.67 ± 0.03	0.73 ± 0.04	0.83 ± 0.04	2.27 ± 0.23	1.98 ± 0.12	1.38 ± 0.08
Docosapentaenoic acid n-3 (DPAn-3)	22:5n-3	1.27 ± 0.02	0.97 ± 0.03	0.94 ± 0.02	1.22 ± 0.03	1.28 ± 0.03	1.15 ± 0.06	1.04 ± 0.04	1.40 ± 0.03	1.25 ± 0.03	1.61 ± 0.11	1.81 ± 0.07	2.11 ± 0.06
Docosahexaenoic acid (DHA)	22:6n-3	4.99 ± 0.06	4.59 ± 0.10	4.38 ± 0.08	5.01 ± 0.09	5.01 ± 0.09	4.66 ± 0.21	4.09 ± 0.11	4.79 ± 0.10	4.98 ± 0.09	5.05 ± 0.23	4.87 ± 0.10	5.33 ± 0.09
Total ω-3 polyunsaturated fatty acids		7.45 ± 0.09	6.94 ± 0.15	6.60 ± 0.11	7.33 ± 0.12	7.51 ±0.14	7.29 ± 0.34	6.61 ± 0.16	7.35 ± 0.15	7.43 ± 0.14	9.44 ± 0.47	9.17 ± 0.24	9.16 ± 0.18
Omega-3 Index (EPA + DHA)		5.83 ± 0.08	5.37 ± 0.13	5.08 ± 0.10	5.73 ± 0.10	5.86 ± 0.13	5.40 ± 0.27	4.76 ± 0.13	5.52 ± 0.13	5.81 ± 0.12	7.32 ± 0.39	6.86 ± 0.20	6.71 ± 0.14
ARA/EPA		14.16 ± 0.55	11.56 ± 0.64	12.40 ± 0.56	13.71 ± 0.52	13.65 ± 0.59	13.64 ± 1.43	12.26 ± 0.58	13.90 ± 0.74	13.78 ± 0.61	5.46 ± 1.26	5.29 ± 0.57	7.13 ± 0.48
ARA/DHA		1.98 ± 0.03	1.62 ± 0.04	1.61 ± 0.03	1.70 ± 0.03	2.00 ± 0.04	1.77 ± 0.07	1.79 ± 0.04	1.86 ± 0.04	1.99 ± 0.04	1.54 ± 0.06	1.44 ± 0.03	1.54 ± 0.03
ω-6/ω-3		4.14 ± 0.07	4.54 ± 0.11	4.64 ± 0.11	3.80 ± 0.09	4.15 ± 0.10	4.33 ± 0.25	4.65 ± 0.14	3.73 ± 0.09	4.27 ± 0.10	3.34 ± 0.30	3.10 ± 0.13	2.77 ± 0.08

Data are % of total fatty acids, presented as means ± SEM.

**Table 6:** Maternal erythrocyte membrane fatty acid composition of cases and controls the day after giving birth.

		Cases		Controls		
		*n* = 24		*n* = 99		
Fatty Acids		Median	(0.25, 0.75)	Median	(0.25, 0.75)	*p*
Total saturated fatty acids		41.73	(40.58, 42.43)	41.08	(39.68, 42.26)	0.345
Total mono-unsaturated fatty acids		23.37	(22.27, 24.55)	23.60	(22.79, 24.82)	0.639
ω-6 polyunsaturated fatty acids						
Linoleic acid (LA)	18:2n-6	14.14	(12.30, 15.97)	14.79	(13.32, 17.43)	0.192
Arachidonic acid (ARA)	20:4n-6	8.67	(7.85, 9.56)	8.35	(7.52, 9.13)	0.304
Docosapentaenoic acid n-6 (DPAn-6)	22:5n-6	0.34	(0.30, 0.37)	0.32	(0.27, 0.38)	0.403
Total ω-6 polyunsaturated fatty acids		26.14	(24.98, 27.40)	26.76	(25.49, 28.15)	0.217
ω-3 polyunsaturated fatty acids						
α-Linolenic acid (ALA)	18:3n-3	0.31	(0.28, 0.38)	0.37	(0.31, 0.44)	0.039
Eicosapentaenoic acid (EPA)	20:5n-3	0.65	(0.53, 0.96)	0.66	(0.51, 0.81)	0.345
Docosapentaenoic acid n-3 (DPAn-3)	22:5n-3	1.24	(0.99, 1.61)	1.23	(1.02, 1.42)	0.801
Docosahexaenoic acid (DHA)	22:6n-3	4.97	(4.37, 6.13)	4.98	(4.45, 5.56)	0.601
Total ω-3 polyunsaturated fatty acids		7.15	(6.37, 8.62)	7.28	(6.57, 8.07)	0.619
Omega-3 Index (EPA + DHA)		5.47	(4.95, 7.02)	5.66	(5.02, 6.37)	0.515
ARA/EPA		11.71	(7.85, 17.09)	13.03	(10.17, 15.95)	0.438
ARA/DHA		1.67	(1.45, 1.87)	1.68	(1.48, 1.90)	0.861
ω-6/ω-3		3.92	(2.84, 4.31)	3.72	(3.24, 4.13)	0.674

Data are % of total fatty acids. *p*-value: Mann–Whitney U-test. Cases had an EPDS score ≥ 9, and controls had an EPDS score < 9.

**Table 7:** Odds ratios (95% confidence intervals) for psychological distress (EPDS score ≥ 9) according to tertile of omega-3 fatty acids of erythrocytes the day after giving birth.

				Tertile of Fatty Acids		
			1 (Low)	2	3 (High)	*p* for Trend
α-Linolenic acid (ALA) 18:3n-3						
	Range		<0.34	0.34–0.42	>0.42	
	Case		14	6	4	
	Control		33	33	33	
	Model 1 ^a^		1.00	0.43 (0.15–1.25)	0.29 (0.09–0.96)	0.030
	Model 2 ^b^		1.00	0.34 (0.11–1.07)	0.23 (0.06–0.84)	0.018
Eicosapentaenoic acid (EPA) 20:5n-3						
	Range		<0.55	0.55–0.74	>0.74	
	Case		8	5	11	
	Control		33	33	33	
	Model 1 ^a^		1.00	0.63 (0.18–2.11)	1.37 (0.49–3.91)	0.509
	Model 2 ^b^		1.00	0.64 (0.19–2.18)	1.36 (0.47–3.91)	0.544
Docosahexaenoic acid (DHA) 22:6n-3						
	Range		<4.67	4.67–5.32	>5.32	
	Case		10	4	10	
	Control		33	33	33	
	Model 1 ^a^		1.00	0.40 (0.11–1.40)	1.00 (0.37–2.72)	1.000
	Model 2 ^b^		1.00	0.37 (0.10–1.40)	1.15 (0.40–3.31)	0.771

^a^ Crude data; ^b^ multivariable models were adjusted for age, pre-pregnancy body mass index, smoking status, alcohol intake, and taking supplements.

## Data Availability

Not applicable.

## Appendix A

**Table A1:** Mean Edinburgh Postnatal Depression Scale score of participants who consumed seafood ≤3 times per week (sub-analysis).

			Observational Study	Intervention Trial		^a^ *p*	^b^ *p*	^c^ *p*
			Historical Control	Perilla Oil Group	Fish Oil Group			
One month after childbirth		*n*	153	81	71			
EPDS	Mean ± SD		5.7 ± 4.2	5.4 ± 3.3	5.5 ± 3.7			
	score ≥ 9	% (*n*)	22.2 (34)	11.1 (9)	22.5 (16)	0.050	1.000	0.079

*p*-value: Fisher’s Exact test for categorical variables; ^a^ *p*: Historical control vs. perilla oil; ^b^ *p*: Historical control vs. fish oil; ^c^ *p*: Perilla oil vs. fish oil. EPDS, Edinburgh Postnatal Depression Scale; SD, standard deviation.

**Table A2:** Mean Edinburgh Postnatal Depression Scale score of participants who consumed 70% or more of omega-3 fatty-acid oil (sub-analysis).

			Observational Study	Intervention Trial		^a^ *p*	^b^ *p*	^c^ *p*
			Historical Control	Perilla Oil Group	Fish Oil Group			
One month after childbirth		*n*	218	84	82			
EPDS	Mean ± SD		5.6 ± 4.0	5.1 ± 3.4	5.3 ± 3.9			
	score ≥ 9	% (*n*)	21.6 (47)	9.5 (8)	23.2 (19)	0.019	0.757	0.021

*p*-value: Fisher’s Exact test for categorical variables; ^a^ *p*: Historical control vs. perilla oil; ^b^ *p*: Historical control vs. fish oil; ^c^ *p*: Perilla oil vs. fish oil. EPDS, Edinburgh Postnatal Depression Scale; MIBS, Mother-to-Infant Bonding Scale; SD, standard deviation.

**Table A3:** Fatty acid compositions of erythrocytes of cord blood.

Erythrocytes of Cord Blood		Observational Study	Intervention Trial			
		Historical Control	Perilla Oil Group		Fish Oil Group	
Fatty Acids		*n* = 193	*n* = 91		*n* = 85	
Total saturated fatty acids		49.24 ± 0.06	49.84 ± 0.07	**	49.99 ± 0.07	**
Total mono-unsaturated fatty acids		16.3 ± 0.07	15.32 ± 0.08	**	15.31 ± 0.08	**
ω-6 polyunsaturated fatty acids						
Linoleic acid (LA)	18:2n-6	3.94 ± 0.04	4.07 ± 0.05		4.12 ± 0.05	*
Arachidonic acid (ARA)	20:4n-6	14.38 ± 0.06	14.5 ± 0.08		13.64 ± 0.10	**,##
Docosapentaenoic acid n-6 (DPAn-6)	22:5n-6	1.00 ± 0.01	1.03 ± 0.02		0.79 ± 0.02	**,##
Total ω-6 polyunsaturated fatty acids		24.85 ± 0.08	25.27 ± 0.10	**	23.71 ± 0.13	**,##
ω-3 polyunsaturated fatty acids						
α-Linolenic acid (ALA)	18:3n-3	ND	ND		ND	
Eicosapentaenoic acid (EPA)	20:5n-3	0.33 ± 0.01	0.32 ± 0.01		0.63 ± 0.03	**,##
Docosapentaenoic acid n-3 (DPAn-3)	22:5n-3	0.78 ± 0.01	0.85 ± 0.02		1.36 ± 0.04	**,##
Docosahexaenoic acid (DHA)	22:6n-3	6.52 ± 0.06	6.26 ± 0.08	*	6.91 ± 0.07	**,##
Total ω-3 polyunsaturated fatty acids		7.64 ± 0.07	7.43 ± 0.10		8.91 ± 0.12	**,##
Omega-3 Index (EPA + DHA)		6.85 ± 0.06	6.58 ± 0.09	*	7.54 ± 0.08	**,##
ARA/EPA		47.7 ± 1.27	46.18 ± 1.50		26.43 ± 1.44	**,##
ARA/DHA		2.24 ± 0.02	2.36 ± 0.04	*	2.00 ± 0.03	**,##
ω-6/ω-3		3.32 ± 0.04	3.47 ± 0.06		2.72 ± 0.05	**,##

% of total fatty acids; data are presented as means ± SEM; *p*-value: one-way analysis of variance followed by Tukey’s test; * *p* < 0.05, ** *p* < 0.01 vs. historical control; ^##^ *p* < 0.01 vs. perilla oil.

**Table A4:** Fatty acid compositions of colostrum.

Colostrum		Observational Study	Intervention Trial			
		Historical Control	Perilla Oil Group		Fish Oil Group	
Fatty Acids		*n* = 208	*n* = 92		*n* = 88	
Total saturated fatty acids		37.21 ± 0.19	37.70 ± 0.28		37.49 ± 0.32	
Total mono-unsaturated fatty acids		41.40 ± 0.18	40.03 ± 0.29	**	40.61 ± 0.30	
ω-6 polyunsaturated fatty acids						
Linoleic acid (LA)	18:2n-6	12.43 ± 0.10	13.05 ± 0.15	**	12.68 ± 0.16	
Arachidonic acid (ARA)	20:4n-6	0.93 ± 0.02	0.86 ± 0.02	*	0.81 ± 0.02	**
Docosapentaenoic acid n-6 (DPAn-6)	22:5n-6	0.10 ± 0.004	0.09 ± 0.004		0.09 ± 0.004	
Total ω-6 polyunsaturated fatty acids		15.73 ± 0.10	16.21 ± 0.15	*	15.68 ± 0.16	#
ω-3 polyunsaturated fatty acids						
α-Linolenic acid (ALA)	18:3n-3	1.04 ± 0.02	1.21 ± 0.02	**	1.07 ± 0.03	##
Eicosapentaenoic acid (EPA)	20:5n-3	0.24 ± 0.01	0.24 ± 0.01		0.28 ± 0.01	*,#
Docosapentaenoic acid n-3 (DPAn-3)	22:5n-3	0.64 ± 0.01	0.59 ± 0.02		0.86 ± 0.03	**,##
Docosahexaenoic acid (DHA)	22:6n-3	1.34 ± 0.03	1.24 ± 0.05		1.32 ± 0.05	
Total ω-3 polyunsaturated fatty acids		3.42 ± 0.06	3.45 ± 0.09		3.68 ± 0.09	*
Omega-3 Index (EPA + DHA)		1.58 ± 0.04	1.48 ± 0.06		1.60 ± 0.06	
ARA/EPA		4.50 ± 0.16	4.56 ± 0.25		3.51 ± 0.20	**,##
ARA/DHA		0.76 ± 0.02	0.77 ± 0.03		0.67 ± 0.02	*,#
ω-6/ω-3		4.84 ± 0.08	4.92 ± 0.11		4.44 ± 0.10	**,##

% of total fatty acids; data are presented as means ± SEM; *p*-value: one-way analysis of variance followed by Tukey’s test; * *p* < 0.05, ** *p* < 0.01 vs. historical control; ^#^ *p* < 0.05, ^##^ *p* < 0.01 vs. perilla oil.
